# Autologous femoral head structural bone grafting in primary total hip arthroplasty for adult developmental dysplasia of the hip: mid-term clinical and radiographic outcomes

**DOI:** 10.1186/s12891-026-09842-6

**Published:** 2026-04-20

**Authors:** Hong-da Wang, Zhi-min Tian, Xing-yu Shan, Huan-xi Li, Chun-nuo He, Rui-ling Xu, Hao-qiang Zhang

**Affiliations:** 1 Department of Orthopedics, The 940th Hospital of the Joint Logistics Support Force of the Chinese People’s Liberation Army, No. 333 South Binhe Road, Lanzhou 7, Gansu, 30050 China; 2 The First School of Clinical Medicine, Gansu University of Chinese Medicine, Lanzhou, 730000 China; 3 Department of Orthopedic Surgery, Heshan People’s Hospital, No.39 Renmin Road, Shaping Street, Heshan, Guangdong 529700 China

**Keywords:** Developmental dysplasia of the hip, Total hip arthroplasty, Structural bone grafting, Acetabular bone defect, Autograft

## Abstract

**Objective:**

To evaluate the clinical and radiographic outcomes of autologous femoral head structural bone grafting in primary total hip arthroplasty (THA) for adult developmental dysplasia of the hip (DDH).

**Methods:**

A retrospective analysis was conducted on 22 patients (30 hips) with DDH who underwent primary cementless THA utilizing autologous femoral head structural bone grafting between August 2014 and July 2024. Perioperative parameters (operative time, intraoperative blood loss), functional outcomes (Harris Hip Score (HHS)), and radiographic outcomes (vertical and horizontal displacement of the hip rotation center relative to the teardrop, limb length discrepancy (LLD) in unilateral cases, Wiberg center-edge (CE) angle, acetabular component coverage, graft coverage) were quantified. Complications were recorded throughout follow-up.

**Results:**

The cohort comprised 5 males and 17 females, mean age 51.73 ± 7.66 years (range: 33–63 years), including 14 unilateral (6 left, 8 right) and 8 bilateral cases. Mean follow-up was 62.4 ± 40.45 months (range: 12–120 months). Mean operative time was 130.67 ± 30.92 minutes (range: 85–195 min); mean intraoperative blood loss was 423.33 ± 253.5 mL (range: 100–1400 mL). The mean HHS improved significantly from 47.77 ± 7.16 preoperatively to 91.20 ± 5.24 at final follow-up (P < 0.0001). Significant restoration of the hip center was achieved: vertical distance decreased from 4.55 ± 1.48 cm to 2.78 ± 0.80 cm, horizontal distance decreased from 6.97 ±1.88 cm to 3.44 ± 0.67 cm (both P < 0.0001). Unilateral LLD was reduced from 25.45 ± 14.05 mm to 5.29 ± 2.91 mm (P ＜ 0.0001). The CE angle increased significantly from 11.53 ± 4.70° to 41.90 ± 4.59° (P < 0.0001). Acetabular component coverage was 96–100% (mean 97.80% ± 1.13%); graft coverage was 19–48% (mean 34.34% ± 7.44%). All grafts demonstrated complete consolidation without collapse or resorption. Surgical incisions healed uneventfully. No complications including periprosthetic infection, loosening, dislocation, or thromboembolic events occurred during follow-up.

**Conclusion:**

In primary THA for adult DDH, autologous femoral head structural bone grafting achieves effective biological reconstruction of the hip center, provides high acetabular component coverage, corrects limb length discrepancy, and significantly restores hip function. The technique demonstrated reliable graft osseointegration and favorable clinical outcomes at early- to mid-term follow-up.

## Introduction

Developmental dysplasia of the hip (DDH) is a developmental disorder characterized by incongruence between the femoral head and acetabulum. This pathoanatomy frequently results in deficient femoral head coverage, varying degrees of subluxation or dislocation, and acetabular bone deficiency. Over time, these biomechanical alterations can lead to severe osteoarthritis, hip pain, functional limitations, and a significant deterioration in lower limb function and quality of life [1, 2]. Total hip arthroplasty (THA) represents one of the most effective interventions for symptomatic DDH in adults. It reliably alleviates hip pain, restores hip function and limb length, and substantially enhances patient quality of life [1, 3, 4]. However, the dysplastic acetabulum in DDH patients frequently presents complex anatomical abnormalities. A critical determinant of successful THA in this population is the reconstruction of an acetabulum that ensures both initial component stability and long-term functional integrity [4, 5].

Contemporary biological acetabular reconstruction techniques include: acetabular component placement at a high hip center [6, 7], the medial protrusio technique (MPT) [7, 8], utilization of small-diameter acetabular components, and structural bone grafting [6]. Each technique confers distinct biomechanical consequences [7]. Among these, structural bone grafting using the autologous femoral head provides enhanced bone stock and superior coverage. This technique facilitates reconstruction of a more anatomical acetabular morphology, restores the hip’s rotational center, enhances the initial stability and long-term survivorship of the acetabular component, and preserves valuable bone stock for potential future revision surgery [9, 10].

Despite these advantages, the technique remains subject to ongoing debate regarding potential limitations. Concerns persist regarding graft resorption, component loosening, and incomplete graft incorporation, which may compromise clinical outcomes. To address these uncertainties, we conducted a retrospective analysis evaluating clinical outcomes following autologous femoral head structural bone grafting in THA for DDH, with the objective of providing evidence-based assessment of this reconstruction method’s efficacy.

## Methods

### Study design and participants

This retrospective study was conducted in strict accordance with the principles of the Declaration of Helsinki and was approved by the Medical Ethics Committee of the 940th Hospital of the Joint Logistics Support Force of the Chinese People’s Liberation Army. This retrospective cohort study evaluated adult patients with DDH who underwent primary THA with autologous femoral head structural bone grafting. Inclusion criteria were as follows: (1) Primary THA for DDH performed in our institution between August 2014 and July 2024; (2) Age ≥ 18 years; (3) Utilization of autologous femoral head structural bone grafting; (4) Cementless prosthesis implantation. Exclusion criteria included: (1) Prior periacetabular osteotomy; (2) Metabolic bone diseases potentially affecting bone metabolism (e.g., nephrotic syndrome, hyperparathyroidism); (3) Concomitant knee joint deformity or severe knee arthritis; (4) Loss to follow-up or incomplete clinical data.

**Fig. 1:**
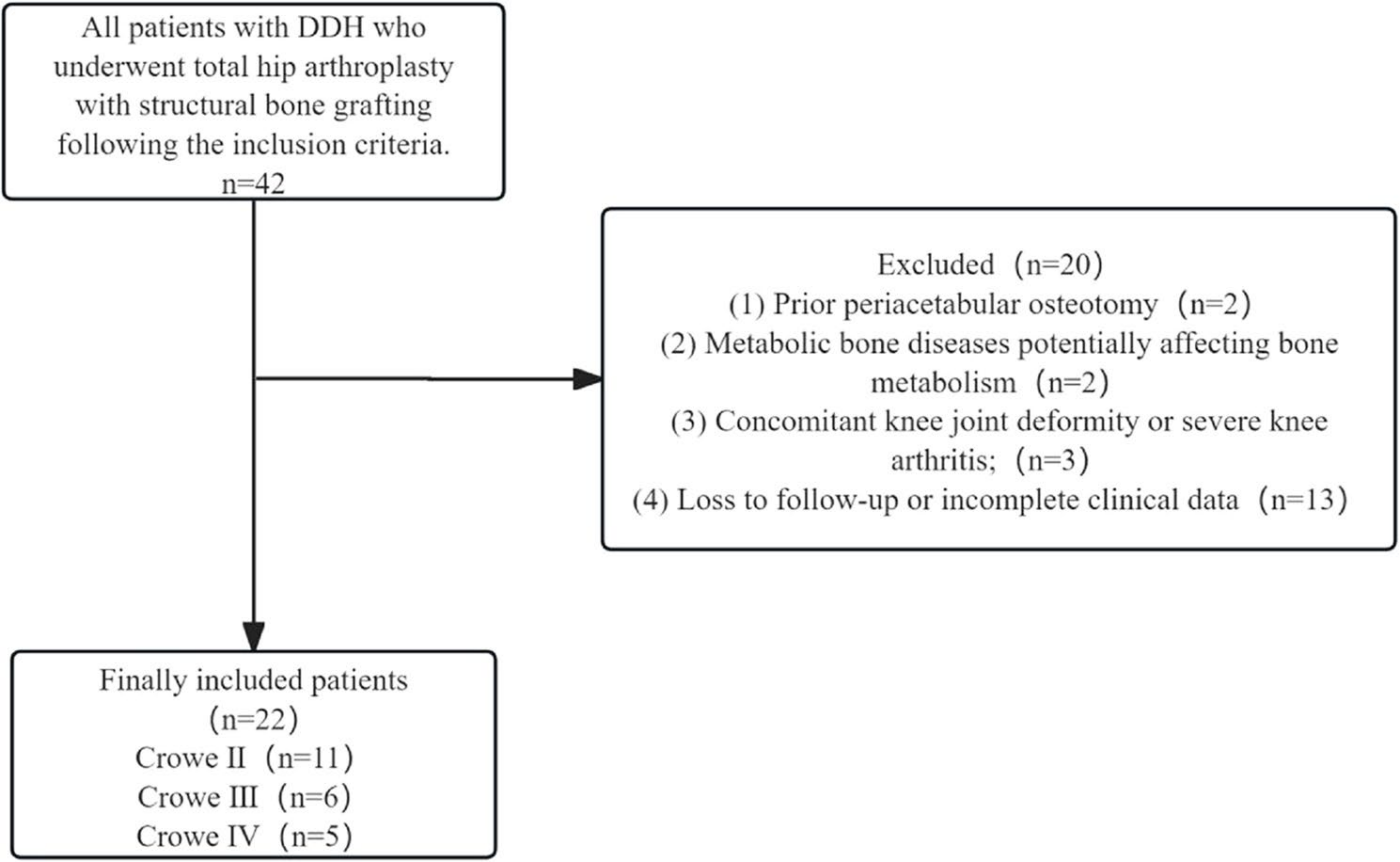
Flowchart of the study

Through the Hospital Information System (HIS), 42 patients with a primary diagnosis of DDH during the study period were initially identified. (Note: Cases where DDH was not the primary diagnosis may have been excluded). Application of inclusion and exclusion criteria yielded a final cohort of 22 patients (30 hips), comprising 5 males and 17 females (Fig. 1). The mean age was 51.73 ± 7.66 years (range: 33–63 years), and the mean body mass index (BMI) was 23.9 ± 4.0 kg/m² (range: 18.6–30.1 kg/m²). All patients presented with preoperative limping, hip pain, and limited joint function. Unilateral involvement was present in 14 cases (6 left, 8 right) and bilateral involvement in 8 cases. The mean preoperative Harris Hip Score (HHS) [11] was 47.77 ± 7.16, and the mean center-edge (CE) angle measured 11.53 ± 4.70°. For unilateral cases, the mean leg length discrepancy (LLD) was 25.45 ± 14.05 mm. Preoperative vertical and horizontal distances of the hip rotation center relative to the teardrop were 4.55 ± 1.48 cm and 6.97 ± 1.88 cm, respectively. According to the Crowe classification [12], distribution was as follows: 11 type II, 6 type III, and 5 type IV cases (Table 1).

**Table 1:** Patient demographic data (*n* = 22)

Indicator	Value
Age (years)	51.73 ± 7.66 (33–63)
BMI (kg/m²)	23.9 ± 4.0 (18.6–30.1)
Gender (Female/Male)	17/5
Crowe Classification	
II	11
Ⅲ	6
IV	5
Laterality (cases)	
Left/Right	6/8
Bilateral	8

*Values are expressed as mean (range)

BMI Body Mass Index

### Preoperative preparation

1. Imaging assessment: Preoperative evaluation included pelvic computed tomography (CT) with 3D reconstruction to assess native acetabular morphology. For complex cases, 3D-printed models were utilized preoperatively to determine optimal acetabular component positioning and the required inferior translation of the hip center. LLD was quantified, and the need for intraoperative femoral shortening osteotomy was assessed.
2. Prosthesis planning: A porous tantalum trabecular metal acetabular component (Zimmer) was selected. Femoral stem selection, based on femoral anatomy, included either a primary stem (CLS® stem, Zimmer) or a dedicated dysplasia stem (S-ROM® [Johnson & Johnson] or Wagner Cone® (Zimmer). Autologous femoral head graft fixation was achieved using 3.5 mm cancellous bone screws (Zimmer).

### Surgical technique

General anesthesia was induced, and patients were positioned laterally. A standard posterolateral approach (Kocher -Langenbeck) was employed. Sequential incisions of the skin, subcutaneous tissue, and fascia lata were made. The gluteus maximus was split bluntly, and the gluteus medius was retracted anteriorly. The external rotator tendons were identified, transected and tagged. The joint capsule was incised to expose the acetabulum and femoral head. Following acetabular assessment, femoral neck osteotomy was performed approximately 1 cm proximally to the lesser trochanter.

The true acetabulum was prepared by excising the labrum and debriding soft tissues. A 38 mm acetabular reamer was used to initiate removal of articular cartilage and sclerotic bone from the acetabular roof. Sequential reaming, guided by intraoperative findings, shaped the acetabular bed. The harvested autologous femoral head was denuded of cartilage and sclerotic bone, then contoured into a hemispherical graft matching the acetabular roof defect. The graft was temporarily secured to the superior acetabular rim with two 2.5-mm Kirschner wires. The acetabulum was then progressively reamed according to the preoperative plan and the identified true acetabulum location. After implanting the acetabular component, the Kirschner wires were replaced with definitive fixation using 3.5 mm cancellous bone screws. Finally, the liner was seated, the femoral stem was implanted, the joint was reduced, stability was assessed, the wound was irrigated, a suction drain was placed, and the incision was closed in layers.

### Perioperative management

1. Infection prophylaxis: Perioperative intravenous second-generation cephalosporin prophylaxis was administered for 24 hours. The drain was removed within 24 hours postoperatively.
2. Multimodal analgesia: Regional nerve blocks were combined with oral nonsteroidal anti-inflammatory drugs (NSAIDs). Parecoxib sodium injections were administered as needed.
3. Thromboembolic prophylaxis: Pharmacological prophylaxis (low-molecular-weight heparin or rivaroxaban) was continued for 5 weeks. Mechanical prophylaxis included intermittent pneumatic compression during hospitalization, supplemented by early initiation of ankle pump exercises.
4. Rehabilitation protocol: Non-weight-bearing exercise was initiated on postoperative day 1 while in bed, and patients began partial weight-bearing walking with a walker at two weeks postoperative. Excessive hip flexion and adduction were avoided for 6 weeks. Full weight-bearing was progressively achieved after 8 weeks, accompanied by progressive abductor strengthening exercises. Strenuous activities were prohibited for 3 months.
5. Follow-up schedule: Sutures were removed at two weeks postoperatively. Follow-up assessments, including X-rays and functional evaluation, were scheduled for 1, 3, and 6 months, and annually thereafter to monitor prosthetic stability and graft integration. Throughout the hospitalization and at each follow-up visit (1, 3, 6 months, and annually thereafter), patients were specifically assessed for any potential complications. This included a review of systems, physical examination, and wound inspection, with any reported symptom or sign being documented as a potential adverse event.

### Outcome measures

Clinical outcomes of the acetabular reconstruction technique were systematically evaluated. Primary outcome measures included:

1. Perioperative metrics: Operative time (skin incision to wound closure) and intraoperative blood loss (calculated as suction volume plus weight of saturated gauze sponges).
2. Functional assessment: The Harris Hip Score (HHS) was used to evaluate hip function preoperatively, at 1 year postoperatively, and at the final follow-up.
3. Radiographic assessment: Postoperative anteroposterior pelvic and lateral hip radiographs taken at 1, 3, and 6 months, and annually thereafter were used to monitor the restoration of the hip rotation center and the CE angle (Fig. 2A). Acetabular component coverage was quantitatively analyzed using the method described by Kim et al. [13] (Fig. 2B), while graft coverage was assessed based on the criteria proposed by Mitsunari Kim et al. [14] (Fig. 2B). Osseointegration was evaluated; the acetabular component was assessed via Moore et al. criteria [15] and was femoral component assessed via Engh et al. criteria [16]. Prosthetic loosening was assessed using the Udomkiat et al. criteria [17], defined as the progression of radiolucent lines or the appearance of a new radiolucent line ≥ 1 mm in width at the 24-month follow-up. Heterotopic ossification was graded according to the Brooker classification system [18]. To ensure the accuracy and comparability of the radiographic measurements, a standardized protocol was strictly followed. All postoperative anteroposterior pelvic radiographs were taken with the patient in a supine position with both lower limbs internally rotated 15° to maximize femoral neck length. A consistent tube-to-film distance of 100 cm was maintained, with the X-ray beam centered on the pubic symphysis. To correct for magnification errors inherent in plain radiography, all linear measurements (e.g., vertical and horizontal displacement, LLD) were calibrated using the known diameter of the implanted femoral head as an internal reference standard. For each radiograph, the measured diameter of the prosthetic femoral head was compared to its actual known size to calculate an individual magnification factor. All linear measurements were then divided by this factor to obtain the true, calibration-corrected values. This method minimizes inter- and intra-observation variability and ensures the reliability of the quantitative data.
  **Fig. 2:**
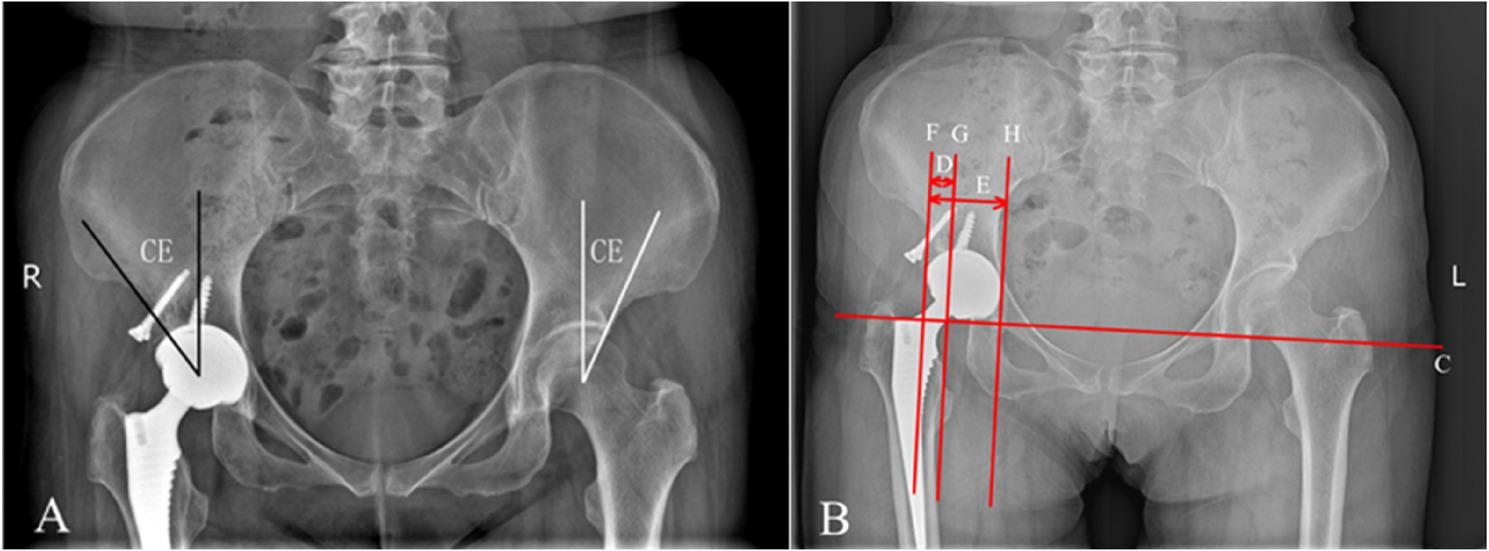
Geometric measurement of the CE angle and calculation of graft coverage. **A** the angle between the vertical line through the femoral head center and the lateral edge of the native acetabulum; the angle between the vertical line through the femoral head center and the lateral edge of the implant. **B** The line connecting the inferior edges of the bilateral teardrops serves as the reference line (line C). Perpendicular lines are drawn from the outermost and innermost edges of the acetabular component to line C, labeled as lines F and H, respectively. The measured parameters include: (1) total prosthesis width (value E): the horizontal distance between lines F and H; (2) graft coverage width (value D): the distance between line F and the perpendicular line from the medial edge of the graft (line G). The graft coverage rate is calculated as: Coverage (%) = (D / E) × 100%. The Acetabular component coverage rate is calculated as: Coverage (%) = (E − D) / E × 100%
4. Complications: All patients underwent systematic monitoring for orthopaedic-specific complications throughout follow-up. Daily wound inspections were performed during hospitalization. At each follow-up visit (1, 3, 6 months, and annually), evaluations assessed hip dislocation, surgical site infection, and neurovascular status. Perioperative and long-term complications were recorded and graded according to the Clavien-Dindo classification [19]. This system categorizes adverse events based on the intervention required: Grade I (deviation from normal postoperative course without need for pharmacological, surgical, endoscopic, or radiological intervention); Grade II (requiring pharmacological treatment beyond Grade I, including blood transfusions or total parenteral nutrition); Grade III (requiring surgical, endoscopic, or radiological intervention); Grade IV (life-threatening complication necessitating intensive care management); and Grade V (death). Minor events (e.g., transient nausea) were recorded; major complications were predefined as Grade III or higher. All events were classified by two independent reviewers, with discrepancies resolved by consensus. All patients completed scheduled follow-ups with no losses. Radiographically, graft collapse was defined as any loss of graft height or structural integrity on serial radiographs; graft resorption was qualitatively assessed; acetabular component loosening was defined as progression of radiolucent lines or a new line ≥ 1 mm at 24 months [17]; femoral component loosening was defined as progressive subsidence ≥ 5 mm or continuous radiolucent lines ≥ 2 mm [16].
5. Radiographic Measurement Reliability: All radiographic measurements, including hip rotation center distances, center-edge angle, LLD, acetabular component coverage, and graft coverage—were performed independently by two experienced orthopedic surgeons blinded to clinical information. Inter-observer reliability was assessed by comparing measurements from both observers for all 30 hips. After a minimum four-week interval, one observer repeated all measurements on a randomly selected subset of 15 hips to assess intra-observer reliability. Intra-class correlation coefficients (ICCs) with 95% confidence intervals were calculated using a two-way random-effects model (absolute agreement) for inter-observer reliability and a one-way random-effects model for intra-observer reliability. ICC values were interpreted as follows: <0.5 indicating poor, 0.5–0.75 moderate, 0.75–0.9 good, and > 0.9 excellent reliability.

### Statistical analysis

Statistical analyses were conducted using SPSS version 25.0 (IBM Corp., Armonk, NY, USA). Normality for all continuous variables was assessed using the Shapiro-Wilk test, which confirmed normal distribution (*P* > 0.05), justifying the use of parametric tests. Data are presented as mean ± standard deviation. Preoperative and postoperative comparisons were analyzed using paired sample t-tests. For primary outcomes, 95% confidence intervals for mean differences and effect sizes (Cohen’s d) were calculated to comprehensively assess clinical relevance. Inter- and intra-observer reliabilities for radiographic parameters were evaluated using ICCs, employing two-way and one-way random-effects models, respectively. Statistical significance was set at two-tailed *P* < 0.05. Subgroup analyses based on Crowe classification (Type II vs. Type III/IV combined) were performed to investigate the impact of disease severity on surgical outcomes. Types III and IV were grouped due to the limited sample size of Type IV (*n* = 5) and their comparable surgical complexity. Preoperative and final follow-up HHS were compared between subgroups using independent samples t-tests. The incidence of major and minor complications was also compared descriptively between subgroups. All analyses adhered to the STROBE guidelines for observational studies. Two independent investigators performed data verification to ensure accuracy and reproducibility.

## Results

This study demonstrated a mean operative time of 130.67 ± 30.92 min (range: 85–195 min) with 423.33 ± 253.5 mL intraoperative blood loss (range: 100–1400 mL). All surgical wounds achieved primary healing without incident.

The reliability of the radiographic measurements was excellent. The ICCs for inter-observer reliability ranged from 0.91 to 0.98 for all parameters (hip rotation center distances, CE angle, LLD, and graft coverage), indicating a high level of agreement between the two independent observers. The intra-observer reliability was also excellent, with ICCs ranging from 0.93 to 0.99, demonstrating high consistency for the repeated measurements by the same observer.

1. Functional outcomes: A significant improvement in functional outcome was observed. The mean HHS increased from 47.77 ± 7.16 preoperatively to 83.20 ± 2.23 at 1 year, preoperatively to 91.20 ± 5.24 at final follow-up (mean difference 43.43 [95% CI 40.51 to 46.35]; t = 30.44, P < 0.0001; Cohen’s d = 5.56).
2. Radiographic parameters: Radiographic evaluation revealed significant restoration in the position of the hip rotation center postoperatively. The vertical distance decreased from 4.55 ± 1.48 cm to 2.78 ± 0.80 cm (mean difference − 1.77 [95% CI -2.27 to -1.26]; t = -7.14, *P* < 0.0001; Cohen’s d = 1.30), and the horizontal distance decreased from 6.97 ± 1.88 cm to 3.44 ± 0.67 cm (mean difference − 3.54 [95% CI -4.26 to -2.82]; t = -10.08, *P* < 0.0001; Cohen’s d = 1.84). In patients with unilateral DDH, the LLD was reduced from 25.45 ± 14.05 mm to 5.29 ± 2.91 mm (mean difference -20.16 mm [95% CI -25.51 to -14.82 mm]; t= -7.85, P < 0.0001; Cohen's d = 1.67). The CE angle improved significantly from 11.53 ± 4.70° to 41.90 ± 4.59° (mean difference 30.37 [95% CI 28.16 to 32.58]; t = 28.09, *P* < 0.0001; Cohen’s d = 5.56) (Table 2). Acetabular component coverage ranged from 96% to 100% (mean: 97.80% ± 1.13%), and graft coverage ranged from 19% to 48% (mean: 34.34% ± 7.44%). At final follow-up, all structural bone grafts demonstrated complete osseointegration without radiographic evidence of collapse or resorption; both acetabular and femoral components remained radiographically stable.
  **Table 2:** Comparison of clinical outcomes

Measure	Preoperative	Postoperative	*P*-value	Mean of differences(95% CI)	Cohen’s d
Harris Hip Score	47.77 ± 7.16	91.20 ± 5.24	*P* < 0.0001	43.43(40.51to 46.35)	5.56
Hip Rotation Center (cm)					
Vertical Distance	4.55 ± 1.48	2.78 ± 0.80	*P* < 0.0001	-1.77 (-2.27 to -1.26)	1.30
Horizontal Distance	6.97 ± 1.88	3.44 ± 0.67	*P* < 0.0001	-3.54 (-4.26 to -2.82)	1.84
LLD (mm)	25.45 ± 14.05	5.29 ± 2.91	*P* < 0.0001	-20.16 (-25.51 to -14.82)	1.67
CE* (°)	11.53 ± 4.70	41.90 ± 4.59	*P* < 0.0001	30.37 (28.16 to 32.58)	5.13

*CE (Wiberg center-edge angle): the angle between the vertical line through the femoral head center and the lateral edge of the acetabulum.95% CI: 95% Confidence Interval of the Difference
3. Complications: The cohort remained free of major complications (Clavien-Dindo grade ≥ III) throughout the perioperative period and follow-up. All patients completed the scheduled follow-up assessments per protocol, with no cases lost to systematic complication monitoring. There were no instances of neurovascular injury, surgical site infection, deep vein thrombosis, acetabular or femoral component loosening, or hip dislocation. All recorded adverse events were minor, with only two patients experiencing transient postoperative nausea that resolved with conservative management. Both acetabular and femoral components exhibited stable fixation, with no radiographic evidence of significant migration, resorption, or femoral stem subsidence (Figs. 3 and 4).
  **Fig. 3:**
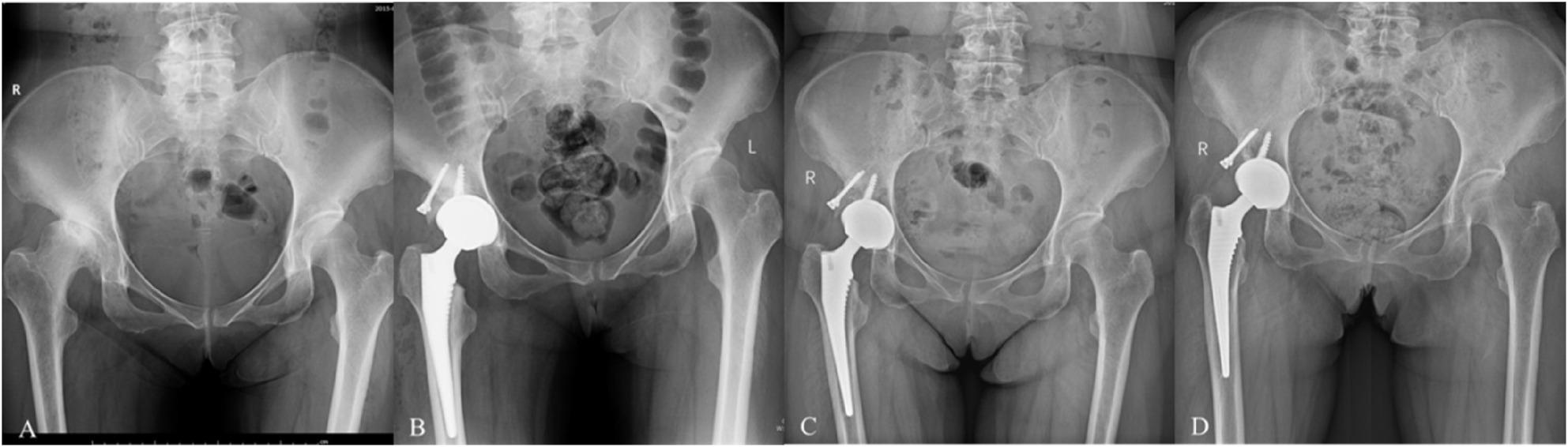
A patient in the early fifties with right-sided DDH, Crowe type II. **A** Preoperative pelvic radiograph shows right acetabular dysplasia with roof defect (CE angle = 10°). **B** Postoperative X-ray demonstrates biological acetabular reconstruction with autologous femoral head structural bone graft, achieving sufficient acetabular component coverage (graft coverage = 23%, acetabular component coverage = 98%). **C** One-year postoperative X-ray shows good graft healing and satisfactory osseointegration at the prosthesis-bone interface (CE angle = 25°). **D** Final follow-up (10 years postoperatively) X-ray reveals stable prosthesis-bone interface without signs of loosening, complete integration of graft and host bone, and maintained graft morphology without significant volume reduction or resorption. LLD improved from 10 mm to 0 mm
  **Fig. 4:**
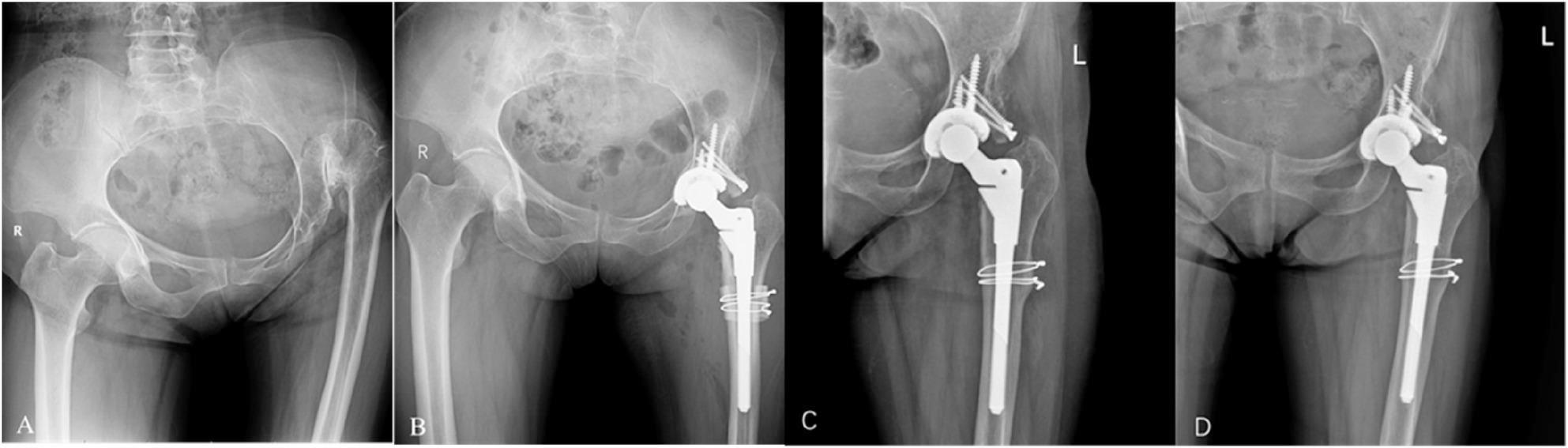
A patient in the late fifties with left-sided DDH, Crowe type IV. **A** Preoperative pelvic radiograph demonstrating left acetabular dysplasia with a superolateral bone defect (CE angle = -5°). **B** Postoperative radiograph showing biological acetabular reconstruction using autologous femoral head structural bone graft, achieving sufficient component coverage (graft coverage = 30%, acetabular component coverage = 96%); concomitant proximal femoral osteotomy fixed with cerclage wiring was also performed. **C** One-year postoperative radiograph confirming satisfactory graft union, stable osseointegration at the prosthesis-bone interface, and bony healing at the osteotomy site (CE angle = 30°). **D** Final follow-up radiograph at 8 years postoperatively shows no signs of acetabular or femoral component loosening, complete graft-host bone integration, and maintained acetabular stability; localized osteolysis is noted around the screw tail, but the overall graft morphology remains well-preserved without significant resorption or collapse. LLD improved from 70 mm to 10 mm
4. Subgroup Analysis: To assess the impact of disease severity, outcomes were compared to Crowe Type II (*n* = 11) and Type III/IV (*n* = 19) hips (Table 3). Although the Crowe III/IV group demonstrated a trend toward poorer preoperative function, the difference in mean Harris Hip Score (HHS) was not statistically significant (47.09 ± 7.91 vs. 48.18 ± 5.92; *P* = 0.68). At final follow-up, both subgroups achieved significant functional improvement, with comparable postoperative HHS (91.47 ± 5.60 vs. 90.73 ± 4.71; *P* = 0.71). No major complications (Clavien-Dindo ≥ III) occurred in either subgroup, and minor adverse events were similarly low. These findings indicate that autologous femoral head structural grafting is equally effective and safe across varying DDH severity.
  **Table 3:** Subgroup analysis of clinical outcomes by crowe classification

Parameter	Crowe Type II (*n* = 11)	Crowe Type III/IV (*n* = 19)	*P*-value
Preoperative HHS	50.36 ± 7.62	46.16 ± 6.59	0.128
Postoperative HHS	90.73 ± 5.24	92.00 ± 4.87	0.508
Major Complications (≥ III)	0	0	1.00

## Discussion

Performing THA in patients with DDH presents significant challenges, predominantly related to acetabular reconstruction. Key issues include acetabular roof and posterosuperior wall bone deficiencies, resulting in insufficient component coverage and potential superior displacement of the hip rotation center [5]. This study demonstrates that THA utilizing autologous femoral head structural bone grafting yields favorable clinical and radiographic outcomes in this cohort with early- to mid-term follow-up. The significant improvement in HHS from 47.77 ± 7.16 preoperatively to 91.20 ± 5.24 at the final follow-up (*P* < 0.0001) reflects a substantial functional restoration. The CE angle increased significantly from 11.53 ± 4.70° to 41.90 ± 4.59° (*P* < 0.0001). Furthermore, high acetabular component coverage (mean 97.80% ± 1.13%), appropriate graft coverage (mean 34.34% ± 7.44%), and universal graft union collectively contributed to excellent initial stability and promising mid-term survivorship.

The outcomes of this technique in our series are consistent with several reports in the literature, although direct comparison is limited by different study designs and follow-up durations. Kim et al. [14] reported a 94% 10-year survivorship using Kaplan-Meier analysis with revision as the endpoint (95% CI: 92–96%) for cementless acetabular components combined with bulk autografts, with 97% (95% CI: 95%–99%) showing no loosening, demonstrating excellent long-term outcomes. Konno et al. [20] further research reported via Kaplan-Meier analysis a 10-year survivorship of 100% (95% CI: 100%) in patients with > 50% graft coverage and 98% (95% CI: 94%–100%) in those with lesser coverage, supporting robust mid- to long-term results. Conversely, Zahar et al. [21] reported less favorable outcomes: at a mean 11.6-year follow-up after bulk structural grafting (cemented or cementless), the aseptic loosening revision rate was 16%, with 84% overall survivorship; Kaplan-Meier analysis indicated significant decline beyond 15 years. In contrast, Maruyama et al. [22], employing combined bulk/impaction grafting with medial-reduced cemented cups, reported only one loosening revision at 10-year mean follow-up. These divergent outcomes suggest implant survivorship variations are primarily associated with several key determinants, including prosthesis fixation method (cemented vs. cementless), the technical sophistication of bone grafting, the anatomical accuracy of component positioning, and the extent of graft coverage.

Consistent with reports by Feng et al. [9] and Kim et al. [14], our mid-term results confirm high graft union rates and sustained implant stability [9, 23, 24]. Furthermore, the absence of perioperative complications, including infection, dislocation, or thromboembolic events supports the technique’s safety. The mean graft coverage (34.34% ± 7.44%) significantly augmented acetabular bone stock and initial component stability. This facilitates early prosthesis-bone osseointegration while mitigating stress concentration on the cup periphery, thereby enhancing long-term fixation. However, concerns persist regarding the load-bearing capacity of structural grafts. Seminal clinical studies from the early 1990s, primarily involving cemented acetabular components, suggested that when a structural autograft supports more than 30%-50% of the acetabular component’s load-bearing surface, the risks of nonunion, graft resorption, and subsequent loosening increase significantly [25, 26]. These observations, though empirically derived, have become important clinical guidelines. It must be acknowledged that these classic thresholds were inferred from mid-term clinical failures and radiographic findings, rather than from direct in vivo biomechanical measurements or prospective validation. Thus, they should be viewed as indicative benchmarks reflecting multifactorial interactions, not absolute biological cut-offs.

Despite a mean graft coverage of 34.34%, theoretically within the “high-risk zone,” all grafts healed completely without collapse or resorption, even at coverage up to 48%. This seemingly paradoxical finding highlights the multifactorial nature of bone healing. While coverage area provides the anatomical foundation, it is not the sole determinant. Initial mechanical stability achieved through cortical screw fixation minimized interfacial micromotion. Concurrently, meticulous preparation of the acetabular bone window and precise contouring of the autogenous femoral head ensured a close host-graft interface. Finally, standardized postoperative bone health management was implemented based on preoperative bone mineral density status. For patients with osteoporosis (T-score ≤ − 2.5), subcutaneous denosumab (60 mg every 6 months) was administered for at least 24 months, combined with oral calcitriol (0.25 µg twice daily) and calcium carbonate with vitamin D₃ tablets (each containing 600 mg calcium and 400 IU vitamin D₃, one tablet twice daily). Non-osteoporotic patients received the same regimen of calcium and calcitriol alone during the postoperative period. Early protected weight-bearing standing provided appropriate mechanical stimulation to the grafted area, promoting graft healing. This multifactorial healing response, supported by contemporary understanding of fracture healing and graft incorporation [27], explains the satisfactory outcomes observed in our series despite exceeding traditional thresholds.

Subgroup analysis further reinforces the robustness of this technique. Despite the greater anatomical distortion and surgical complexity in Crowe Type III and IV hips, postoperative functional outcomes (mean HHS 92.00 ± 4.87) were statistically indistinguishable from those in Type II hips (90.73 ± 5.24, *P* = 0.508). This suggests that meticulous execution, including accurate cup placement in the true acetabulum and stable screw fixation, can overcome challenges posed by high dislocation, yielding comparable benefits across DDH severity. It supports the principle that anatomical reconstruction of the hip center, when achievable, is a beneficial and attainable goal regardless of initial deformity.

In the context of THA for adult DDH, various acetabular reconstruction techniques have been described, including high hip center placement, the medial protrusio technique (MPT), use of small-diameter components, and structural bone grafting. In the present study, autologous femoral head structural grafting achieved prosthetic coverage ranging from 96% to 100%, while MPT has been reported to achieve coverage ranging from 80% to 100% [28]. This technique also enabled biological acetabular reconstruction, as reflected by significant improvement in the CE angle. Anatomical reconstruction of the acetabulum and hip rotation center may help avoid issues associated with non-anatomical hip center placement, such as abductor insufficiency or residual limb length discrepancy [1, 29, 30], for Crowe type II to IV dysplasia, augmentation of bone stock using autologous graft preserves autogenous tissue that may be valuable in potential future revision surgeries. Zirvecan et al. [31] reported a 10-year survivorship of 96.8% in 31 DDH hips at a mean follow-up of 13.5 years using Kaplan-Meier analysis, supporting the long-term durability of this approach. Other techniques, such as MPT, involve a controlled fracture of the medial wall, while high hip center placement, though useful in selected complex cases, represents a biomechanical alteration from the native hip center.

Similarly, metal augments are a valuable tool, particularly for addressing severe segmental defects in revision settings [32]. Biomechanical studies indicate they can provide initial stability comparable to structural grafts for roof defects [33]. However, in primary DDH THA, their application must be weighed against the benefits of biological reconstruction. While augments offer off-the-shelf convenience and avoid concerns about graft resorption, they do not restore bone stock and their removal in a future revision could be challenging and potentially create larger defects. Therefore, the choice between structural grafting, metal augments, or other techniques is not a matter of inherent superiority, but rather a decision guided by individual patient anatomy, defect morphology, surgeon expertise, and the philosophical goal of either biological reconstruction or prosthetic replacement of bone. Autologous structural grafting remains a powerful, biologically driven option, particularly for younger patients where bone stock preservation is paramount, though it is technically demanding and requires sufficient autograft quality and quantity. Conversely, metal augments provide a predictable solution when autograft is insufficient or when maximum immediate mechanical stability is prioritized.

This study observed no instances of lower limb neuropathy or hip dislocation. K. Gustke et al. [34] identified excessive nerve traction secondary to limb lengthening as a primary cause of post-THA neuropathy in DDH. Historically, lengthening > 4 cm was considered a significant risk factor [35, 36]. Contemporary approaches, incorporating intraoperative neuromonitoring and advanced rehabilitation, now permit greater lengthening in carefully selected cases to correct discrepancy without substantially increasing neurological risk [35]. Dislocation risk is predominantly governed by component positioning and soft tissue balance [37]. In our cohort, meticulous soft tissue balancing via precise limb length adjustment, combined with accurate acetabular component placement within the true acetabulum achieving optimal coverage and orientation strictly within the Lewinnek safe zone [38], collectively contributed to the absence of dislocations.

## Conclusion

In conclusion, this study confirms that structural bone grafting utilizing the autologous femoral head is an effective technique in primary THA for adult patients with DDH. This technique reliably achieves graft-host osseointegration, reconstructs native acetabular anatomy, and provides sufficient prosthetic coverage with initial stability, yielding favorable clinical and radiographic outcomes at early- to mid-term follow-up. Recent technological advancements, including 3D-printed guides, artificial intelligence (AI)-enhanced preoperative planning, and robotic-assisted surgery are propelling acetabular reconstruction toward unprecedented precision, individualization, and functionality restoration. The integrated application of these innovations holds significant promise for further enhancing osseointegration, optimizing long-term implant survivorship, and improving functional prognosis. This evolution may ultimately offer DDH patients increasingly reliable, durable, and personalized therapeutic solutions.

### Study limitations

This investigation has several inherent limitations: (1) The mean follow-up of 62.4 ± 40.45 months represents only early- to mid-term outcomes; consequently, the study lacks sufficient data to assess long-term implant survivorship, late graft resorption, or aseptic loosening beyond 10 years. While our results are encouraging, extended surveillance is required to confirm durability, particularly given previous reports of late failures beyond 10–15 years. (2) The retrospective design and limited cohort size constrain statistical power and generalizability, precluding multivariate regression to control for potential confounders such as age, sex, and Crowe classification. (3) Radiographic assessment relied on plain radiographs without systematic CT-based three-dimensional analysis, potentially limiting precision in evaluating graft integration and residual bone stock. (4) Despite standardization efforts, technical bias may exist due to single-surgeon involvement and prostheses from multiple manufacturers. (5) The absence of patient-reported outcome measures (PROMs) restricts comprehensive evaluation from the patient’s perspective, as the Harris Hip Score alone cannot capture pain perception, activity participation, or satisfaction. Future studies should incorporate validated PROM instruments for a more holistic assessment.

Notably, all procedures adhered rigorously to standardized clinical protocols, with implant selection and surgical approach following predefined criteria to mitigate technical heterogeneity. Future research should prioritize larger-scale, multi-center prospective studies that ideally incorporate randomized controlled designs, along with standardized surgical protocols and implant selection, to validate the generalizability and long-term reliability of these findings. Such studies should include sufficient sample sizes to allow for adjustment of confounding factors, including age, sex, and disease severity, thereby drawing more robust conclusions.

## Data Availability

The datasets analyzed during the current study are available from the corresponding author on reasonable request.

## Abbreviations

DDH: Developmental dysplasia of the hip
THA: Total hip arthroplasty
MPT: Medial protrusio technique
LLD: Leg length discrepancy
HHS: Harris Hip Score
BMI: Body Mass Index
95% CI: 95% Confidence Interval
